# Attentional Resources Are Needed for Auditory Stream Segregation in Aging

**DOI:** 10.3389/fnagi.2017.00414

**Published:** 2017-12-22

**Authors:** Elizabeth Dinces, Elyse S. Sussman

**Affiliations:** ^1^Department of Otorhinolaryngology, Albert Einstein College of Medicine, Bronx, NY, United States; ^2^Department of Neuroscience, Albert Einstein College of Medicine, Bronx, NY, United States

**Keywords:** selective attention, auditory scene analysis, event-related potentials (ERPs), mismatch negativity (MMN), automatic processing

## Abstract

The ability to select sound streams from background noise becomes challenging with age, even with normal peripheral auditory functioning. Reduced stream segregation ability has been reported in older compared to younger adults. However, the reason why there is a difference is still unknown. The current study investigated the hypothesis that automatic sound processing is impaired with aging, which then contributes to difficulty actively selecting subsets of sounds in noisy environments. We presented a simple intensity oddball sequence in various conditions with irrelevant background sounds while recording EEG. The ability to detect the oddball tones was dependent on the ability to automatically or actively segregate the sounds to frequency streams. Listeners were able to actively segregate sounds to perform the loudness detection task, but there was no indication of automatic segregation of background sounds while watching a movie. Thus, our results indicate impaired automatic processes in aging that may explain more effortful listening, and that tax attentional systems when selecting sound streams in noisy environments.

## Introduction

Adults over age 60 with normal peripheral auditory functioning often report having difficulty listening in noisy environments. An important function of the auditory system is to sort the deluge of sounds typically entering the ears, separating the mixture of sounds emitted from different sources. This process of auditory stream segregation (Bregman, 1990) enables us to listen to a single voice in a noisy restaurant or to follow the melody of the cello in an orchestral suite. Research in younger adults suggests that auditory processes automatically segregate the sounds even when the sounds are task irrelevant and are ongoing in the background (Sussman et al., 1999; Pannese et al., 2015). This automatic stage of segregation, based on the spectro-temporal characteristics of the input, is necessary as it facilitates the ability to focus attention (a limited resource) to a single stream to get meaning from within it (e.g., the words in a speech stream). In this way, automatic auditory processes contribute to the ability to navigate noisy situations by providing a first level of structure on the sounds.

Focusing attention to the sounds can also influence the stream segregation process (Sussman et al., 2005; Sussman and Steinschneider, 2009). For example, selectively attending to a subset of sounds can promote stream segregation at smaller frequency differences between tones than would occur if the same set of tones were ignored and task-irrelevant (Sussman et al., 1998, 1999; Sussman and Steinschneider, 2009). Thus, attention can be used to refine the stream segregation process, interacting with the automatic level of processing, and influence the organization and maintenance of sound representations held in auditory memory (Sussman et al., 2002, 1998). These observations in younger adults led to our hypothesis of the current study that impaired automatic processing impacts on the ability to identify sound events when there are competing background sounds.

Studies investigating auditory stream segregation ability in aging individuals with normal hearing status have found that stream segregation abilities differ as a function of age (Amenedo and Diaz, 1998; Grimault et al., 2001; Bertoli et al., 2002, 2005; Snyder and Alain, 2006, 2005; He et al., 2008), and have implicated impaired early, automatic processes associated with such functions as concurrent vowel segregation (Snyder and Alain, 2005; Getzmann et al., 2015), gap detection (Alain et al., 2004), frequency discrimination (Bertoli et al., 2005), and build-up to stream segregation (Ezzatian et al., 2015) but have not conclusively identified what may be contributing to the observed impairments. These studies have not compared effects of active and passive listening on target detection, and therefore have not been able to pinpoint the level of the deficit observed in the behavioral performance. To do this in the current study, we measured brain responses during passive and active listening to address how attentive and automatic processes interact and contribute to impaired performance.

Auditory event-related brain potentials (ERPs), which are time-locked to specific stimulus events and extracted from the ongoing EEG record, can index sound processing for attended and unattended inputs. Specifically, the mismatch negativity (MMN) is an auditory-specific component (Nyman et al., 1990), that reflects memory processes (Javitt et al., 1996), and does not require directed attention to the sounds to be elicited (Näätänen et al., 2001; Sussman et al., 2003; Winkler et al., 2005). Thus, MMN is ideally suited for probing auditory sensory memory of unattended sound input. The P3b is a non-modality specific component that indexes attentional processes, such as target detection. Both MMN and P3b are elicited by detected sound violations (Squires et al., 1975; Näätänen et al., 1978). However, the MMN can be elicited irrespective of the direction of attention (during passive or active listening), whereas the P3b requires attention to be focused on the sounds to be elicited.

In the current study, we measured ERPs and behavioral responses during listening to a mixture of high and low frequency sounds to compare passive (watching a movie) and active (press the key for the louder sounds) listening processes associated with stream segregation in aging individuals with normal hearing status. The goal was to identify the level of the processing difficulty (bottom–up or top–down) that may be contributing to deficits in identifying target sounds when there are competing backgrounds sounds, and gain a better understanding of the factors that may be influencing difficulty with stream segregation in noisy environments. Our hypothesis is that impaired automatic processing impacts on the ability to identify sound events when there are competing background sounds. This hypothesis predicts that larger frequency separations would be needed between sounds to automatically segregate streams than would be required when attention is focused on a subset of sounds to actively segregate the sounds and perform a task. Larger frequency separations to automatically segregate a set of sounds would indicate decreased bottom-up processing when attention is directed away from the sounds toward another aspect of the sensory input. A limitation of automatic processing would contribute to impaired ability to actively identify sound events. That is, if the sounds do not automatically segregate, this would increase the need for attentional resources to focus on a subset of sounds and segregate them from the background noise. Thus, attentional resources would be used for accurate stream segregation in aging individuals when automatic stream segregation does not occur. Such impairments could, at least in part, explain why processing sensory input in complex listening environments is more effortful in older individuals with normal peripheral functioning.

## Materials and Methods

### Participants

Eighteen aging adults between the ages of 60–83 years were recruited into the study through flyers posted around the Einstein campus and local area senior centers. The study protocol followed the Code of Ethics of the World Medical Association (Declaration of Helsinki) and was approved by the Internal Review Board of the Albert Einstein College of Medicine. Participants gave written informed consent after the study was explained to them, and were paid for their participation. Sixteen individuals passed a hearing screen (≤30 dB HL for 500, 1000, 1500, 2000, 3000, and 4000 Hz, and type A tympanograms) performed in a sound attenuated and electrically shielded booth. All participants scored 0–1 errors on the Short Portable Mental Status Questionnaire (SPMSQ) (Pfeiffer, 1975). One participant’s data was excluded due to excessive EEG artifact in both sessions. Fifteen participants (4 males, ranging in age from 61–81, *M* = 68 years, *SD* = 6) (11% Asian, 28% African American, and 61% Caucasian) were included in the study. 13 participants completed the study in two sessions occurring on separate days. Two participants completed one session each.

### Stimuli

**FIGURE 1:**
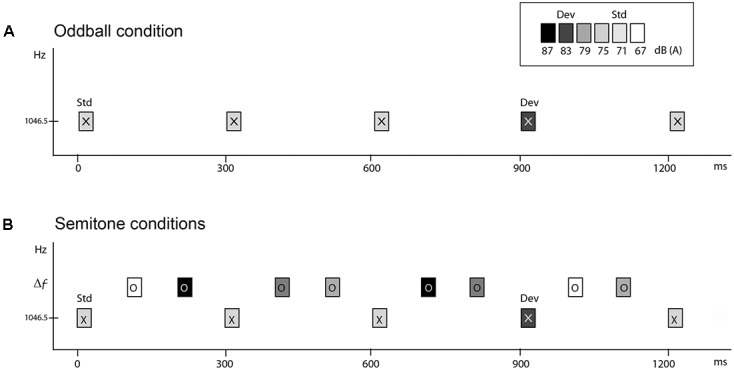
Schematic of the stimulus paradigm. Rectangles marked ‘X’ and ‘O’ represent tones. Shading of the rectangle indicates the intensity value of the tones. The abscissa displays time in milliseconds. The ordinate indicates the frequency scale (in hertz). **(A)** Oddball condition. ‘X’ tones presented at 300 ms onset-to-onset, with the standard presented at 71 dB SPL and the deviant 12 dB higher, 10% and randomly occurring. **(B)** Semitone conditions. Two ‘O’ tones intervene between each of the ‘X’ tones, with randomly varying intensity values, thus presented with a 100 ms onset-to-onset pace. ‘O’ tones were presented in separate conditions, at 1, 5, 11, or 19 ST higher than the 1046.5 Hz ‘X’ tones (Δ*f*). The task was to press the response key when the deviant (dev) intensity tones were detected amongst the standard (Std) ‘X’ tones.

All stimuli were pure tones, 50 ms in duration (7.5 ms rise and fall time) created with Neuroscan Sound 2.1 software (Compumedics, Charlotte, NC, United States), and presented binaurally via E-A-RTONE 3A insert earphones (Indianapolis, IN, United States). Tones were calibrated at peak-to-peak equivalents in SPL using a 2209 Brüel & Kjær sound-level meter (Norcross, GA) with a Brüel & Kjær 4152 artificial ear. Tones had a frequency of 1046.5 Hz (called ‘X’ tones) and 1, 5, 11, or 19 semitones (ST) distance from the X tone (1108.7, 1396.9, 1975.5, or 3135.9 Hz, respectively, called ‘O’ tones) (**Figure 1**).

### Electroencephalographam (EEG) Recordings

Electroencephalographam (EEG) was recorded using a 32-channel electrode cap (Electro-Cap International INC, Eaton, OH) in the modified 10–20 international configuration Additional electrodes were placed on the left and right mastoids (LM and RM, respectively). An external electrode placed at the tip of the nose was used as the reference electrode. A bipolar montage between the FP1 electrode and an external electrode placed under the left eye recorded the vertical electro-oculogram (EOG). A bipolar configuration between F7 and F8 recorded the horizontal EOG. Impedances were maintained below 5k Ω. EEG and EOG were digitized at a sampling rate of 1000 Hz (0.05–200 Hz bandpass) using a Neuroscan Synamps amplifier, (Compumedics Corp., Raleigh, NC, United States).

### Experimental Procedures

To obtain comparison between passive and active listening in auditory stream segregation, we presented five conditions in each of two separate sessions (one for active and one for passive listening), on separate days (occurring between 7–21 days apart). The five conditions presented in each session were as follows. One condition presented the X tones alone (Oddball condition) at 300 ms SOA (**Figure 1A**). The Oddball condition served as a baseline measure for MMN elicitation to intensity deviants when there were no competing sounds. There were four semitone (ST) conditions (1, 5, 11, and 19 ST) in which both ‘X’ and ‘O’ tones were presented with a stimulus onset asynchrony (SOA) of 100 ms in a fixed pattern (XOOXOO) (**Figure 1B**). The larger the frequency separation between X and O tones, the more likely two distinct frequency streams would be perceived (Bregman, 1990; Carlyon et al., 2001; Sussman and Steinschneider, 2009; Sussman et al., 2015). In all conditions, 88% of the X tones had an intensity of 71 dB SPL and randomly, 12% of X tones had an intensity of 83 dB SPL. Intervening O tones had one of four different intensity values (67, 75, 79, or 87 dB SPL) randomly distributed with equal probability. Importantly, the intensities of the O tones spanned above and below the tone intensity values of the X tones, so that the X tones could not be detected as the highest or the lowest intensity tones in the ST sequences. Thus, frequency was used to cue stream segregation and intensity was used for deviance detection.

### MMN Control Conditions

To delineate the MMN, the standard ERP waveform was subtracted from the deviant ERP waveform. We ran five “control” conditions in which the intensity values of the standard and deviant X tones were reversed. This was done to subtract the waveforms from two physically matched stimuli (i.e., the 83 dB X tone when it was the louder deviant tone in the block was subtracted from the 83 dB X tone when it was the louder standard tone in the control block).

The stimulus sequences were presented in three minute blocks of 1534 tones for the ST conditions and 1011 tones for the Oddball condition, and the order of blocks was randomized across participants in both *Passive* and *Active* sessions. In each block, the first 10 tones were excluded from the analysis to avoid any orienting responses evoked by the onset of the stimulus run being added into the grand averages. 1524 *control ‘X’* tones were collected for each of the ST conditions and 1494 *control ‘X’* tones were obtained for comparison for the Oddball condition.

### Session Tasks

Participants sat in a sound attenuated and electrically shielded booth. During EEG recording in the Active session, subjects performed the Loudness Detection Task. Participants listened to the sounds, and were instructed to focus on the lower-pitched sounds, to ignore the higher-pitched sounds, and to press a response key whenever they detected an oddball intensity deviant tone in the lower frequency stream (Loudness Detection Task, **Figure 1**). In effect, they were trying to segregate out the lower frequency sound stream from the higher frequency sound stream. In the Oddball condition, participants performed the loudness detection task, with X tones occurring at the same stimulus rate as they occurred in the ST conditions, but there were no competing sounds to ignore. Practice was provided prior to the Active session. In the practice session, the oddball sequence was used to demonstrate the task, and a larger, 23 ST condition was used to demonstrate stream segregation and provide practice for the loudness detection task. The 23 ST practice condition was not presented during the main experiment.

In the *Passive* session, participants were instructed to ignore the sounds presented to their ears and watch a closed-captioned movie played without sound. After this recording session, the cap was removed and a behavioral measure of the global perception of the sounds as one or two streams was obtained (Global Perception task). Participants were instructed to listen to sounds in each of the four ST conditions and report whether they heard the sounds ‘more like’ one or ‘more like’ two sound streams. A practice was given to provide instructions and examples of the task. A larger 23 ST frequency separation was used to provide an example of what two streams might sound like and a 1 ST frequency separation was used to demonstrate what one stream might sound like. Sound sequences were shorter than during the recording session (15 s each), with 20 trials for each of the four ST conditions presented randomly (80 trials altogether). At the end of each 15 s sound sequence, participants reported whether the sequence sounded “more like” one or two streams and the experimenter recorded the responses. The Global Perception task lasted approximately 12 min. A second task was used to obtain a behavioral measure of loudness detection (Loudness Detection task), in which participants pressed a response key when they identified the louder or softer tone in the oddball sequence as was done during the EEG recording during the *Active* session (described above).

The sessions were randomly assigned, the first session for half of the participants was *Passive* listening, and the first session for the other half was *Active* listening. Breaks were given as needed, with at least one longer break about midway in which participants were unhooked from the amplifier to walk around and have a snack. Total session time including breaks was approximately 88 min for the *Passive* session and 84 min for the *Active* session.

## Data Analysis

### Behavioral Data

For the Global Perception task, the proportion of trials perceived as “two streams” for each ST condition was calculated for each participant separately. For the Loudness Detection task, mean reaction time (RT), mean hit rate (HR), and mean false alarm rate (FAR) were calculated separately for each participant in each condition (**Table 1**). The response window for correct responses was set from 30–900 ms from stimulus onset of a deviant tone. Reaction time was calculated as the average time in milliseconds from the onset of the deviant stimulus. Hit rate was calculated as the number of correct button presses to the deviant tone divided by the number of total targets. False alarms were button presses to any tone other than the intensity deviant tones. To provide a more conservative estimate of the FAR, due to the rapid pace of the stimuli, FAR was calculated using the number of possible response windows as the base estimate to calculate FAR rather than the number of total non-target stimuli as the denominator (Bendixen and Andersen, 2013). This will be denoted as the ‘adjusted false alarm rate’ (**Table 1**). Separate one-way repeated-measures analysis of variance (ANOVA) was used to statistically determine differences among conditions for the Global Perception and Loudness Detection tasks.

**Table 1:** Behavioral data.

	Global Perception Task	Loudness Detection Task		
Condition	Proportion	Hit rate	*Adjusted*	Reaction time
	‘two-streams’		False alarm rate	(ms)
Oddball	-	0.85 (0.21)	0.02 (0.03)	336 (73)
19ST	0.85 (0.22)	0.75 (0.24)	0.003 (0.002)	379 (83)
11ST	0.77 (0.23)	0.68 (0.22)	0.09 (0.16)	404 (99)
5ST	0.33 (0.33)	0.23 (0.23)	0.14 (0.26)	498 (76)
1ST	0.16 (0.22)	0.17 (0.22)	0.23 (0.31)	-

*Standard deviation in parenthesis. – no response*.

### ERP Data

Electroencephalographam was filtered offline using a finite impulse response (FIR) filter with a bandpass of 0.5–30 Hz (zero phase shift, 24 dB/octave rolloff) using Neuroscan 4.5 Edit software (Compumedics Corp., Raleigh, NC, United States). The filtered EEG was then segmented into 600 ms epochs, including a 100 ms pre-stimulus period, averaged separately by stimulus type [deviants (DV) and control standards CS)] in the active and passive sessions. Epochs were baseline corrected before artifact rejection was applied with a criterion set at ±75 μV on all electrodes (EOG and EEG). On average, 18% of the overall epochs were rejected due to artifact. The remaining EEG epochs were averaged separately by stimulus type and then baseline corrected to the pre-stimulus period.

The MMN was delineated in the grand-averaged difference waveforms (deviant-minus-control standard). The mastoid electrode was used to identify MMN peak latency in the grand-averaged difference waveforms because of overlap with the N2b component at Fz electrode that occurs when attending to target sounds in the *Active* session. The P3b peak was identified at the Pz electrode where the signal-to-noise-ratio is greatest (**Figure 3**) in the *Active* session. To statistically evaluate the presence of the MMN component, the data were re-referenced to the left mastoid. A 50 ms interval for MMN and a 60 ms interval for P3b (centered on the peak latency of the component, **Tables 2**, **3**) were used to obtain mean amplitudes for the standard and deviant ERPs, in each individual, separately in each condition. For conditions in which no component could be visually identified, the interval was used from the smallest ST condition where it was statistically present (**Tables 2**, **3**).

**Table 2:** Mismatch negativity (MMN) component mean latency and amplitude.

Active session		Passive session		
Condition	Peak latency	Amplitude	Peak latency	Amplitude
	(ms)	(μV)	(ms)	(μV)
Oddball	125 (16)	-2.60 (1.90)^∗∗^	122 (16)	-2.01 (1.83)^∗^
19ST	149 (18)	-1.64 (1.65)^∗∗^	158 (16)	-0.43 (1.22) n.s.
11ST	159 (27)	-1.08 (1.42)^∗^	187 (18)	-0.59 (1.44) n.s.
5ST	243 (14)	-0.51 (0.95) n.s.	243 (18)	0.30 (0.46) n.s.
1ST	242 (16)	-0.43 (0.67) n.s.	249 (19)	0.01 (0.60) n.s.

*^∗^p < 0.05, ^∗∗^p < 0.01, n.s. = not significant. Standard deviation in parentheses.*

**Table 3:** P3b component.

Condition	Peak latency	Amplitude
	(ms)	(μV)
Oddball	401 (21)	3.61 (3.18)^∗^
19ST	408 (19)	2.06 (2.72)^∗^
11ST	467 (20)	2.63 (2.35)^∗^
5ST	505 (20)	0.68 (1.74) n.s.
1ST	508 (16)	0.04 (0.91) n.s.

*^∗^p < 0.01, n.s. = not significant. Standard deviation in parentheses.*

To confirm the presence of MMN and P3b, one-sample one-sided *t*-tests were performed at the electrode of greatest signal to noise ratio. For those conditions in which ERP components were present, repeated-measures ANOVA with factors of condition (Oddball, 19, 11, 5, 1 ST) and scalp distribution (Fz, Cz, Pz) were used to compare amplitude and latency.

For all statistical analyses, degrees of freedom were corrected using Greenhouse–Geisser estimates of sphericity where data violated the assumption of sphericity, and corrected *p* values are reported. For *post hoc* analyses, when the omnibus ANOVA was significant, Tukey HSD *post hoc* analyses for repeated measures were used and contrasts were reported as significantly different at *p* < 0.05. All statistical analyses were performed using Statistica 12 software (Statsoft, Inc., Tulsa, OK, United States).

## Results

### Behavioral Data

**Table 1** presents the behavioral data for both experiments. For the Global Perception task, there was a main effect of condition (*F*_3,39_ = 33.36, 𝜀 = 0.77, *p* < 0.001, $\eta_{p}^{2}$ = 0.72), which *post hoc* calculations showed was due to significantly higher report of two stream perception for the 19 and 11 ST conditions than the 5 and 1 ST conditions. There was no significant difference between 19 and 11 or between 5 and 1 ST. In the Deviance Detection task, HR was higher with larger frequency separations, and highest for the Oddball condition (main effect of condition on HR, *F*_4,52_ = 44.03, 𝜀 = 0.39, *p* < 0.001, $\eta_{p}^{2}$ = 0.77). *Post hoc* calculations showed this effect was due to a significantly higher HR for deviants in the Oddball, 19 and 11 ST conditions than the 5 and 1 ST conditions. There were no significant differences among Oddball, 19, and 11 ST conditions, and between 5 and 1 ST conditions.

Comparing RT for the conditions in which the deviant could be sufficiently detected (Oddball, 19, and 11 ST conditions), RT was shortest in the Oddball condition, with no significant difference in RT between the 19 and 11 ST conditions (main effect of condition: *F*_2,26_ = 11.66, 𝜀 = 0.66, *p* < 0.001, $\eta_{p}^{2}$ = 0.47).

False alarm rate (FAR) was largest for the 1 ST condition compared to the Oddball and 19 ST conditions (main effect *F*_4,52_ = 4.9, 𝜀 = 0.44, *p* = 0.02, $\eta_{p}^{2}$ = 0.27), with no differences among the other conditions (**Table 1**).

### Event-Related Potentials

**FIGURE 2:**
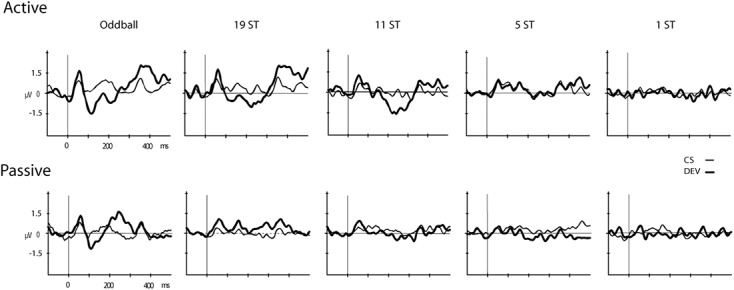
Event-related brain potentials (ERPs). Grand-mean ERPs elicited by the deviant (DEV, thick solid line) and the control standard (CS, thin solid line) are displayed (Fz electrode) for the Active **(Top)** and Passive **(Bottom)** sessions for all conditions (Oddball, 19 ST, 11 ST, 5 ST, and 1 ST). The abscissa shows the timing in milliseconds and the ordinate displays amplitude in microvolts.

**FIGURE 3:**
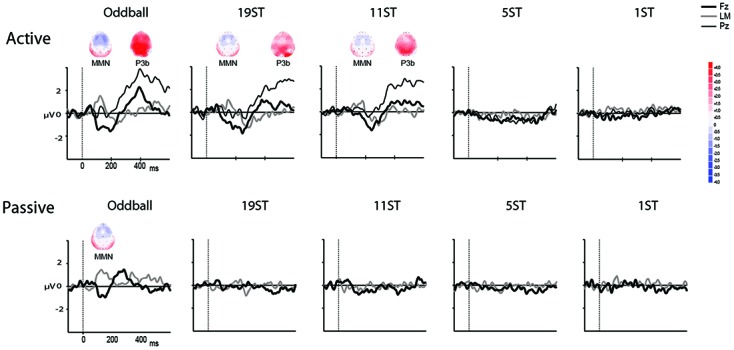
Difference waveforms. Grand mean difference waveforms (deviant-minus-standard ERPs displayed in **Figure 2**) at Fz (thick black line), Pz (thin black line), and the left mastoid (LM, thick gray line) in each of the conditions for the Active **(Top)** and Passive **(Bottom)**. Time is shown in milliseconds on the abscissa and amplitude is indicated in microvolts on the ordinate. Significantly elicited ERP components are labeled, and displayed along with maps showing the scalp voltage distribution at the peak latency of each component. Blue represents negative polarity and red represents positive polarity.

**Figures 2**, **3** display the ERP waveforms for the passive and active sessions, respectively. **Tables 2**, **3** present the mean amplitudes and standard deviations for the MMN and P3b components, respectively.

#### Mismatch Negativity

Mismatch negativities were significantly elicited at Oddball, 19 ST, and 11 ST conditions in the *Active* session but only in the Oddball condition of the *Passive* session. MMN amplitude elicited by intensity deviants in the Oddball conditions in the *Active* vs. *Passive* sessions were not significantly different (*t*_13_ = 1.2, *p* = 0.28) (**Table 2** and **Figure 2**). In the *Active session*, there was a smaller amplitude MMN elicited by deviants in the ST conditions compared to the Oddball condition. MMN amplitude in the Oddball condition was significantly larger (more negative) than the MMN elicited in the 11 ST condition, but no different than the 19 ST condition (main effect of condition: *F*_2,26_ = 4.94, 𝜀 = 0.89, *p* = 0.019, $\eta_{p}^{2}$ = 0.28). The scalp distribution was consistent with known MMN topography, with fronto-central minima (larger at Fz and Cz than Pz) (main effect of electrode: *F*_2,26_ = 15.47, 𝜀 = 0.76, *p* < 0.001, $\eta_{p}^{2}$ = 0.54).

#### P3b

P3b was significantly elicited in the Oddball, 19 ST, and 11 ST conditions in the *Active* session (**Table 3** and **Figure 3**). There was no amplitude difference among the P3b components elicited (*F*_2,26_ = 2.07, *p* = 0.15). P3b components were not elicited in the *Passive* session when attention was directed to watching a movie.

## Discussion

Our results show a dramatic difference in auditory stream segregation ability between passive and active listening conditions in aging individuals with normal peripheral functioning. There was no physiological indication of stream segregation during passive listening. Intensity deviants were apparently not detected and no MMN was elicited at any frequency separation (19, 11, 5, or 1 ST) in the Passive Listening conditions. This indicates that stream segregation did not occur automatically at these frequency separations. MMN was elicited, however, in the Oddball condition by intensity deviants during passive listening (while subjects watched a movie) when there were no competing background sounds. For comparison, in younger adults, we have found only a small difference between passive and active indices of physiological stream segregation: MMN was elicited with a 7 ST Δ*f* during passive listening and with a 5 ST Δ*f* when actively segregating sounds (Sussman and Steinschneider, 2009). Thus, the current results suggest that the automatic system is not operating as efficiently in aging as in younger individuals.

We also found that when actively segregating out a single frequency stream (the low tones), the ability to identify intensity deviants occurred at slightly larger frequency separations than found for younger adults doing the same task: 11 ST during active listening in older adults (current study) and 5 ST during active listening in younger adults (Sussman and Steinschneider, 2009). Impaired automatic processing may increase the need for attentional resources to focus on a set of sounds and segregate them from the background noise, to refine the stream segregation process.

These results showing impaired ability to automatically segregate sounds may therefore explain why older individuals with normal peripheral functioning have decreased ability to select individual sound events in noisy environments: the automatic processes are not segregating sounds to a degree that would allow attention to operate more efficiently. Our results support the hypothesis that more attentional resources are required in aging individuals to segregate sound input. When the automatic system is not supporting sound segregation, attentional resources are needed, first to aid in segregating the sounds (not accomplished by automatic processes), and then to perform the target identification task. In younger adults, stream segregation is not automatic at 5 ST, attentional resources are needed to segregate the sounds and then the within-stream loudness deviants can be detected (Sussman and Steinschneider, 2009). Therefore, the absence of MMN in the Active 5 ST condition in aging, in the current study, indicates an aging-related limitation.

Results from the two behavioral tasks (Global Perception and Loudness Detection) were consistent with each other and with the ERP results. When participants’ reported hearing two streams at 19 and 11 ST (Global Perception Task) they had a high HR to intensity deviants (Loudness Detection Task) in those conditions. Moreover, MMN was elicited by intensity deviants when subjects reported hearing two streams in the 19 and 11 ST conditions. At the smaller frequency separations (5 and 1 ST conditions), there was no indication of stream segregation from the behavioral responses in either of the behavioral tasks, and no MMNs were elicited by deviants in those conditions. Thus, ERP results were consistent with the perception of stream segregation.

Mismatch negativities were elicited by intensity deviants in the Oddball conditions in both sessions (passive and active listening). However, no specific attention effect found. The amplitude and latency of the MMN component did not vary as a function of the direction of attention. That is, attention did not enhance detection of the oddball intensity deviants at this large difference between the intensity standard and intensity deviant (Δ12 dB). Moreover, this results indicates that the absence of MMN in the *passive* ST conditions cannot be attributed to inability to passively detect the intensity deviant, or to any effect due to actively listening for the intensity deviant. The absence of MMN is more likely attributed to an inability to automatically segregate out the two frequency streams (up to Δ 19 ST) in the passive conditions, and to a limit on attention resources for processing within-stream events in the active conditions. These results also indicate that attention is not globally impaired in aging. Attention breaks down during complex listening conditions when automatic systems are not performing optimally.

The results of this study add to our understanding of why noisy situations provide a listening challenge in aging. Background sounds appear to interfere with automatic segregation processes that would facilitate target detection in the attended stream. In the Active session, the attentive system facilitated the segregation of the sounds, allowing the standard-deviant relationship to be detected (Δ12 dB), and MMNs were elicited by intensity deviants. However, aging adults needed larger frequency separations even to actively segregate high from low tones and perform the deviance detection task compared to younger adults in similar listening situations (Sussman and Steinschneider, 2009). Stream segregation ability is thus reduced at both automatic and attentive levels of processing, contributing to greater challenges for perceiving distinct sound sources in noisy environments.

A limitation of the current study is that we do not include a control group of younger adults. However, our comparison group of younger adults is taken from results of a previously published study (Sussman and Steinschneider, 2009). This study used similar ST conditions in Active and Passive listening conditions (1, 5, 7, 11 ST), presented stimuli through the same setup (hardware and software), recorded EEG with the same amplifiers and in the same recording booth (same laboratory) as the group of older adults reported in the current study. Thus, the results are generally comparable. In addition, we are in process of conducting a similar study, which will replicate and extend the current findings to a wider range of age groups, spanning across younger and older adults.

To summarize the main findings of the current study, automatic stream segregation was not effective in aging adults. There was no indication of automatic stream segregation at any of the ST distances tested. Impaired automatic processing may increase the need for attentional resources to segregate sounds from background noise, thus taxing attentional resources that would otherwise be used to identify sound events occurring within the individual sound streams. Our results thus indicate that more effortful listening is required in complex listening environments to offset deficient automatic processes. Such impairments may therefore explain why older individuals with normal peripheral functioning have decreased ability to select sound events in noisy environments: the automatic processes are not segregating sounds to a degree that would allow attention to operate efficiently.

## Author Contributions

ES designed the study, ED collected the data, ES and ED analyzed the data, interpreted the results, and wrote the manuscript.

## Conflict of Interest Statement

The authors declare that the research was conducted in the absence of any commercial or financial relationships that could be construed as a potential conflict of interest.
